# Coupling of terminal differentiation deficit with neurodegenerative pathology in Vps35-deficient pyramidal neurons

**DOI:** 10.1038/s41418-019-0487-2

**Published:** 2020-01-06

**Authors:** Fu-Lei Tang, Lu Zhao, Yang Zhao, Dong Sun, Xiao-Juan Zhu, Lin Mei, Wen-Cheng Xiong

**Affiliations:** 1 Department of Neuroscience and Regenerative Medicine, Medical College of Georgia, Augusta University, Augusta, 30912 Georgia; 2 Department of Neurosciences, Case Western Reserve University, School of Medicine, Cleveland, OH 44106 USA; 3 Key Laboratory of Molecular Epigenetics of Ministry of Education, Institute of Cytology and Genetics, Northeast Normal University, Changchun, Jilin 130024 China

**Keywords:** Lysosomes, Neuroscience, Neurological disorders

## Abstract

Vps35 (vacuolar protein sorting 35) is a key component of retromer that regulates transmembrane protein trafficking. Dysfunctional Vps35 is a risk factor for neurodegenerative diseases, including Parkinson’s and Alzheimer’s diseases. Vps35 is highly expressed in developing pyramidal neurons, and its physiological role in developing neurons remains to be explored. Here, we provide evidence that Vps35 in embryonic neurons is necessary for axonal and dendritic terminal differentiation. Loss of Vps35 in embryonic neurons results in not only terminal differentiation deficits, but also neurodegenerative pathology, such as cortical brain atrophy and reactive glial responses. The atrophy of neocortex appears to be in association with increases in neuronal death, autophagosome proteins (LC3-II and P62), and neurodegeneration associated proteins (TDP43 and ubiquitin-conjugated proteins). Further studies reveal an increase of retromer cargo protein, sortilin1 (Sort1), in lysosomes of Vps35-KO neurons, and lysosomal dysfunction. Suppression of Sort1 diminishes Vps35-KO-induced dendritic defects. Expression of lysosomal Sort1 recapitulates Vps35-KO-induced phenotypes. Together, these results demonstrate embryonic neuronal Vps35’s function in terminal axonal and dendritic differentiation, reveal an association of terminal differentiation deficit with neurodegenerative pathology, and uncover an important lysosomal contribution to both events.

## Introduction

Neurodegenerative diseases, including Alzheimer’s disease (AD), Parkinson’s disease (PD), and frontotemporal dementia (FTD), occur as a result of complex brain degenerative processes with a progressive loss of neurons or brain structures [1]. Among numerous risk factors for neurodegenerative diseases, genetic mutations are believed to be a primary “hit” [2]. While each disease has its unique risk genes, mutations in *VPS35* have been associated with PD and AD [3–5], suggesting that Vps35 dysfunction may be a general risk factor for neurodegenerative disorders.

Vps35 is a key component of retromer that selectively sorts transmembrane proteins/cargos to the trans-Golgi network or plasma membrane [6–8]. Several lines of evidence suggest that Vps35 dysfunction is linked with PD and AD. Vps35 is reduced in the hippocampus of AD patients [9]. Vps35-deficiency increases production of Aβ [9–11] and impairs mitochondrial dynamics and function [12, 13]. Vps35-deficient mice exhibit partial AD- or PD-relevant deficits [10, 11, 14] and overexpressing Vps35 fully recovers the AD phenotype in 3xTg mice [15]. In addition, retromer cargo proteins including APP [16], TREM2 [17], and sortilin1-related receptor (SorLA) [18] are genetic risk factors for AD. Interestingly, sortilin1 (Sort1) and SorLA are members of vacuolar protein sorting ten family receptors. Sort1 has been emerged as a co-receptor in cell death or neurodegeneration regulated by ligands of progranulin (PGRN) and implicated in the pathogenesis of FTD [19]. These observations implicate that Vps35-deficiency may be a common pathological mechanism of neurodegenerative disorders, including AD, PD, and possibly FTD. However, how Vps35-loss results in different neurodegenerative disorders and whether Vps35-loss increases FTD development remains largely unknown.

Vps35 is highly expressed in developing pyramidal neurons [20] as well as dopamine (DA) neurons [14]. We have previously shown that selectively knocking out (KO) Vps35 in DA neurons results in early onset PD-relevant deficits [12]. Here, we investigated Vps35’s function in mouse developing pyramidal neurons. Vps35-KO in embryonic cortical pyramidal neurons results in dendritic maturation defects and axonal spheroid formation. Mice that selectively depleted Vps35 gene in embryonic (by Neurod6-Cre) pyramidal neurons display FTD-like neuropathology, including progressive reduction of cortical thickness, elevation of cortical neuronal death, accumulations of P62, LC3-II, Tdp43, and ubiquitin-conjugated protein levels, impairments in lysosomal morphology and acidification, and reactive gliosis. Further mechanical studies demonstrate an increase of Sort1 in lysosomal compartments of Vps35-KO neurons. Suppression of Sort1 expression by shRNA diminishes Vps35-loss-induced dendritic defects. Expression of lysosomal Sort1 fusion protein in embryonic pyramidal neurons impairs lysosomal functions and recapitulates Vps35-loss-induced deficits. These observations thus uncover Sort1 as a critical cargo of Vps35 to underlie Vps35’s function in developing neurons. Our results suggest that the dysfunctional Vps35-Sort1 pathway in developing pyramidal neurons acts as a detrimental factor not only for neuronal terminal differentiation but also for neurodegenerative pathology.

## Methods and materials

### Animals

Mice were cared according to animal protocols approved by the Institute of Animal Care and Use Committees at the Augusta University and Case Western Reserve University, according to the National Institute of Health (NIH) guidelines. All mice were housed in standard conditions with food and water provided and maintained on a 12 h dark/light cycle. The noon of a day when a vaginal plug is found is designated as E0.5. Experiments were replicated at a minimum of three times with mice derived from independent litters. *Vps35* loxp flanked exon 6 mice have been previously described [12]. *Vps35-cKO* mice were generated by breeding floxed *Vps35* (*Vps35*^*f/f*^) with *Neurod6-Cre* (kindly provided by Dr KA Nave [21]) mice. *Neurod6-Cre* expression alone did not have a detectable effect on the phenotypes described in this manuscript. Thus, the *Cre*-positive or *Cre*-negative littermates were utilized as controls unless indicated otherwise. *Ai9* (stock No. 007907) and *Thy1*-YFP (stock No. 003782) mice were purchased from the Jackson laboratories. For all experiments, mutants were compared with littermate controls. All phenotypic characterizations of *Vps35*-cKO (*Vps35*^*Neurod6*^) and their control mice were in C57BL/6 background. Both male and female mice were used in all experiments.

### Plasmids

The *pCAG-GFP* and *pCAG-Cre* plasmids were purchased from Addgene. To generate the p*CAG-Cre-2A-GFP or BFP* plasmid, the *P2A* domain was added to the C terminus of Cre, then followed by *GFP* or *BFP* cDNA. Their *Cre* efficiency was confirmed by IUE of the plasmid into Ai9 reporter mice.

The *Sort1*-ShR expression vector was generated by the pSuper vector system, the target sequence is 5′-gcacctgacaacaaatggg (*Sort1* shR2#) and 5′-ccgtcctatcaatgtgatt (*Sort1* shR 3#).

To generate *Lyso-Sort1 (CD63-Sort1)* plasmid, the *CD63* cDNA was cloned from mouse whole brain mRNA by TA cloning. *Sort1* cDNA (without the signal peptide) was then inserted into the first luminal loop of *CD63* by Ligation Independent Cloning (LIC) with Exonuclease III. Finally, the OFR with CD63-Sort1 were inserted into a mammalian expression vector with a *CAG* promoter. To test the pH in lysosomes, Lyso-pHluorin (Plasmid #70113) were obtained via Addgene, and the promoter CMV was replaced with *CAG*.

### In utero electroporation (IUE)

IUE was performed as previously described [22]. In brief, pregnant mice were deeply anesthetized with 2% isoflurane (Piramal Healthcare) through anesthesia system (Kent Scientific), and embryos were exposed at E14.5 or E18.5. The plasmid (at a final concentration of ~1 μg/μl) was mixed with fast green (0.1 mg/ml, Sigma-Aldrich), which were microinjected into the lateral ventricle of embryos using a glass capillary. The embryos were electroporated with five 50 ms pulses at 36 V (for E14.5) or 45 V (for E18.5) with a 950 ms interval through ECM-830 (BTX, Holliston, MA). The uterine horns were then gently reinserted into the abdominal cavity and the abdomen wall and skin were sutured. The pups were fixed by cardiac perfusion with 4% paraformaldehyde in 0.1 M phosphate buffer, pH 7.4. at different postnatal days. Their brains were removed and soaked in the fixative for 2–4 h. After rinsing with phosphate-buffered saline (PBS), their coronal vibratome sections (50–100 μm in thickness) were prepared by (VT1000s, Leica) and used for immunostaining or immunohistochemical staining analyses.

### Antibodies for immunohistochemistry and immunoblotting

The following antibodies were used: Mouse monoclonal anti-beta-Actin (diluted 1:5,000; Abcam, ab8227); goat polyclonal anti-aldolase C (diluted 1:200; Santa Cruz, sc-12065); rabbit monoclonal anti-CD11b (diluted 1:200; Abcam, ab133357); rabbit monoclonal anti-cleaved caspase-3 (Asp175) (D3E9) (diluted 1:400; Cell Signaling, 9579); mouse monoclonal anti-GM130 (diluted 1:1000; BD Bioscience, 610822); mouse monoclonal anti-GFAP (1:500; Millipore, MAB360); rabbit polyclonal anti-GFP (1:2,000; Thermo Fisher Scientific, A-11122); rabbit polyclonal anti-EEA1 (diluted 1:5000; Abcam, ab50313); goat polyclonal Iba1 (diluted 1:500; Abcam, ab5076); rat monoclonal anti-Lamp1 (1:500; DSHB, 1d4b); rabbit polyclonal anti-LC3B (diluted 1:500; Abcam, ab48394); mouse monoclonal anti-NeuN (diluted 1:1000; EMD Millipore, MAB377); mouse monoclonal anti-P53 (diluted 1:800; abcam, ab26); Sheep polyclonal anti-PGRN (diluted 1:500; R&D Systems, AF2557); rabbit polyclonal anti-Sort1 (diluted 1:500; Abcam, ab16640); mouse monoclonal anti-P62 (diluted 1:500; Abcam,) ab56416, rabbit polyclonal anti-TDP43 (C-terminal) (diluted 1:500; Proteintech, 12892–1-AP,); rat monoclonal anti-phospho-TDP43 (Ser409/Ser410) (diluted 1:100; Millipore Sigma, MABN14); mouse monoclonal anti-ubiquitin (diluted 1:500; Santa Cruz, sc-8017); mouse monoclonal anti-γH2AX (phosphorylation of the Ser-139 residue of the histone variant H2AX) (diluted 1:500; abcam, ab26350); rabbit polyclonal anti-Vps35 and rabbit polyclonal anti-reticulum 3 (Rtn3) antibody was generated as described previously [10, 23] (diluted 1:2000).

### Immunohistochemistry

Mice of the appropriate age were anesthetized and perfused transcardially with 4% paraformaldehyde/PBS as previously described [10, 12, 14]. Brains were dissected, postfixed, and mounted in agarose prior to vibratome sectioning. Overall, 35–100 μm free-floating vibratome sections of brains were collected in PBS, blocked with 5% normal serum in PBS with 0.5% Triton X-100 and incubated with primary antibodies in blocking solution overnight at 4 °C. P14 vibratome sections were blocked with 5% normal serum in PBS with 0.1% Triton X-100 and 2% DMSO, and incubated with primary antibodies for 3 days. After 1–3 h incubation with Alexa Fluor-conjugated secondary antibodies (Invitrogen or Jackson Immunoresearch) followed by DAPI staining (0.1 μg/ml, Life Technologies), the stained slices were imaged using a confocal laser-scanning microscope (Zeiss LSM510, LSM710 or Nikon A1 plus).

### Image acquisition and analysis

Confocal images of regions of interest were collected from individual brain sections for each animal. For all morphological analyses, images were coded using computer-generated random number sequences at the time of data acquisition and all analyses were performed blinded to the experimental condition. Control and experimental group neurons which were to be directly compared were imaged with the same acquisition parameters.

Z-stack images were collected with ×10 or ×20 objectives and tiled together to generate high-resolution images of whole brain sections. The acquired images were processed using the Zen (Zeiss) Representative images have been cropped and adjusted for brightness and contrast in Photoshop for presentation. For the quantitative analysis of intracellular levels of P62, p-TDP43, Sort1 and pHluorin, the entire neuron was selected, and the fluorescence intensity was measured directly with Image J after a threshold application.

### Dendritic arborization

Individual neurons from *Thy1*-YFP mice or IUEed brain were acquired on a Nikon A1 plus confocal system using a W-Plan Apochromat 25×/1.0 IR-corrected water immersion objective. On average, Z-stacks were acquired from 50 to 70 μm range with 1 μm steps. Dendritic reconstructions were performed using NeuronStudio. A total of 12 control and 12 experimental neurons from three mice each were reconstructed for dendritic analysis. Apical and basal dendrite polarity was quantified by NeuronJ from reconstructed images. Sholl analysis calculated the number of dendrite intersections and dendritic lengths as previously described [24]. Sholl quantification was conducted with an initial 10-μm soma radius and 10-μm concentric radial steps.

### Dendritic spines and microglia imaging and 3D reconstructions

For quantitative analysis of the morphology of dendrite spines and dendrites. Images of dendritic spines/microglia were acquired on a Nikon A1 confocal microscope with a Plan Apo VC 60 X Oil DIC N2 objective (N.A. = 1.4), at 3X zooms, 0.5 um Z-interval, and 1024 × 1024 pixels. Three-dimensional (3D) reconstructions were performed, and spine density was quantified using the Filament module of IMARIS software (Bitplane) as described previously [25, 26].

For the spine/dendrite ratio analysis, secondary branches with similar width of apical dendrites of L2–3 pyramidal neurons in the somatosensory cortex were examined. Spine density was calculated by dividing the total spine number by the dendritic branch length. Spine subtypes were classified based on previously defined morphological criteria [25] and quantitated as follows: (a) mushroom: the spine head diameter is ≥1.5× the diameter of spine neck (dh/dn ≥ 1.5); (b) stubby: head and neck of the spine are approximately of same width, and spine length is not significantly longer than the head diameter (dh/dn < 1.5, L/dh < 2); (c) thin: diameters of spine head and neck are nearly equal, and spine length is greater than spine width (dh/dn < 1.5, L/dh ≥ 2); Only images (blind-coded) with sufficient quality to clearly identify and measure spine shapes were used in the subtype classification, and 20–30 consecutive spines on the same dendrite were analyzed per image.

### Nissl staining, Golgi staining, and TUNEL assay

For Nissl staining sections were incubated in 1% cresyl violet solution for 3–4 min, then washed and dehydrated in increasing ethanol concentrations. The cortical thickness was measured in Nissl-stained sections using ImageJ software. For each animal, we took three measurements in the somatosensory cortex and the visual cortex (*n* = 3–4 animals per genotype). Golgi staining was performed by using the FD Rapid GolgiStain Kit (FD NeuroTechnologies) as described previously [27]. Freshly dissected brain was immersed in the kit’s impregnation solution for 2 weeks. The frozen tissue was sectioned at 150 μm on a microtome, followed by staining according to the manufacturer’s procedures. For the neuronal morphometric analysis, pyramidal neurons were randomly selected from somatosensory cortical layer II/III. Images were acquired (2 or 0.2 μm pitch) using a light microscope at ×10 or ×40 magnification (BX-43; Olympus), and the dendrites and dendritic spines were traced using NeuronStudio software. The total dendritic length, Sholl analysis, and spine density were also calculated by Neuron J (ImageJ). The spinal density was obtained from the apical dendrites within the region 50–150 μm from the soma (total length 1300–1800 μm). The TUNEL assay was used to detect dead cells with DNA fragmentation using Click-iT Plus TUNEL assay (Invitrogen) by following manufacture’s protocol.

### NeuroSilver Staining

Silver staining was used to visualize degenerating neuronal elements in brain sections of Vps35^Neurod6^ mice and age-matched control mice [28]. Sections were processed using the FD NeuroSilver kit (FD Neurotechnologies Inc, Baltimore, MD) according to the manufacturer’s instructions.

### Jade C Staining

Fluoro-Jade C (Jade C) a polyanionic fluorescein derivative that can sensitively and selectively bind to degenerative neurons [29]. To visualize degenerating neurons, brain sections from perfused P14 WT and Vps35^Neurod6^ brains were mounted on gelatin-coated slides and subjected to Jade C staining, according to manufacturer’s protocol (Fluoro-Jade® C, Millipore). Sections were imaged with a Zeiss LSM510 confocal microscope.

### Western blotting

Tissues were dissected from both mutant and littermate control mice and lysed with 5× volume per weight 1% Triton X-100 buffer (1% Triton X-100, 20 mM Tris pH 7.4, 150 mM NaCl, 10% glycerol, protease and phosphatase inhibitor cocktail (Millipore)) by homogenization, incubated on ice-water slurry for 20 min, frozen and thawed twice, and ultracentrifuged at 100,000 *g*, 4 °C for 30 min as described previously [12] and the supernatant taken as the soluble fraction. The Triton-insoluble pellets were sonicated with 2× volume per original sample weight in urea-lysis buffer (7 M urea, 2 M thiourea, 4% CHAPS, and 30 mM Tris, pH 7.5, and a protease and phosphatase inhibitor cocktail (Millipore)) and centrifuged at 100,000 *g* for 30 min at 22 °C. These supernatants were taken as the insoluble fraction. Protein concentration was measured by BCA assay (Pierce).

Lysosomal proteins were isolated as described previously [30]. In brief, neocortex lysates from P1 mice were homogenized in a homogenizing buffer (HM buffer; 0.25 M sucrose, 1 mM EDTA, and 10 mM Hepes, pH 7.0), and then centrifuged at 1500 *g* at 4 °C for 10 min to remove the nuclei and intact cells. Postnuclear supernatants were then subjected to ultracentrifugation through a Percoll density gradient using an ultracentrifuge. An ultracentrifuge tube was layered with 2.5 M sucrose, 18% Percoll in HM buffer, and supernatant (top). The centrifugation was performed at 90,000 *g*, 4 °C, for 1 h. Samples were fractionated into light, medium, and heavy membrane fractions. Heavy membrane fractions contained concentrated bands of cellular organelles and were further layered over a discontinuous iodixanol gradient, generated by mixing iodixanol in HM buffer with 2.5 M glucose (in vol/vol; 27, 22.5, 19, 16, 12, and 8%) and with osmolarity maintained at 300 mOsm for all solutions. After centrifugation at 4 °C for 2.5 h at 180,000 *g* (44,200 rpm), each sample was divided into 12 fractions (0.5 ml each) and the first two fractions were used for further analyses. Cell surface proteins were isolated as previously [31] by using pierce cell surface protein isolation kit.

Overall, 10–20 μg of total protein was loaded for each sample into 8 or 14% gels. Gels were transferred onto Nitrocellulose Blotting Membranes (Bio-rad). Antigen-specific primary antibodies were incubated overnight at 4 °C and detected with species-specific far-red fluorescent-labeled secondary antibodies (Li-Cor). Band quantification was performed using ImageJ software (version 1.44; NIH). Bands of interest were normalized to beta-actin or GAPDH for a loading control.

### Cell line and transfection

Neuro-2a cells (ATCC, RRID: CVCL_0470) were maintained in DMEM medium (Invitrogen) supplemented with 10% FBS at 37 °C and 5% CO2. DNA transfection was performed using a standard polyethyleneimine protocol. They were authenticated based on morphology, and DNA staining revealed no mycoplasma contamination.

### Cultured cortical neuron preparation

Primary dissociated cortical neuronal cultures were prepared from E14.5 mouse embryos, according to protocols previous used for hippocampal neurons [27, 32, 33], with the added step of straining the cells through a Falcon Cell Strainer (Thermo Fisher Scientific Cat# 08–771–1). Mouse cortical neurons were plated on poly-L-lysine-coated coverslip at 50,000–80,000 cells/cm^2^, in Neurobasal medium (Invitrogen, Carlsbad, CA) containing B-27 (Invitrogen), 2 mM Glutamax-I (Invitrogen), and 2.5% FBS (HyClone, Logan, UT). On the second day in vitro (DIV 2), medium was half changed to medium without FBS. Neurons were fixed with 4% PFA, 4% sucrose in PBS for immunocytochemistry at DIV 10–12. Immunostaining of neuronal cultures was performed as described previously [10, 12, 14].

### Quantitative real-time RT−PCR (qRT–PCR)

Total RNA from both mutant and littermate control mice brain was extracted using TRIzol reagent (Life Technologies) as previously described [34, 35]. After homogenizing the samples with TRIzol reagent, chloroform was added and the aqueous layer containing the RNA was isolated and precipitated with isopropanol. Reverse transcription into cDNA was carried out by using the iScript cDNA Synthesis Kit (Cat. #: 170–8890, Bio-RAD) according to manufacturers’ instructions. qRT–PCR was performed with a Bio-Rad CFX96 Real-Time system using Maxima SYBR Green/ROX qPCR Master Mix (Thermo Fisher Scientific). Samples were run in triplicates and all signals were normalized to GAPDH intensity.

### Statistics

Although no statistical methods were used to predetermine sample size, our sample sizes are based on our previous experiences or publications [36–39]. All data are presented as mean ± SEM. GraphPad Prism 8 (GraphPad Software) was used for statistical analysis. Data distribution was assumed to be normal, but this was not formally tested. The two-tailed unpaired *t*-test was used to evaluate statistical significance of two groups of samples. One-way analysis of variance (ANOVA) with a Tukey post hoc test was used to evaluate statistical significance of three or more groups of samples. The *n*-numbers can be found in the figure legends. Statistical significance was defined as *P* < 0.05.

## Results

### Requirement of Vps35 in embryonic pyramidal neurons for axonal and dendritic terminal differentiation

**Fig. 1:**
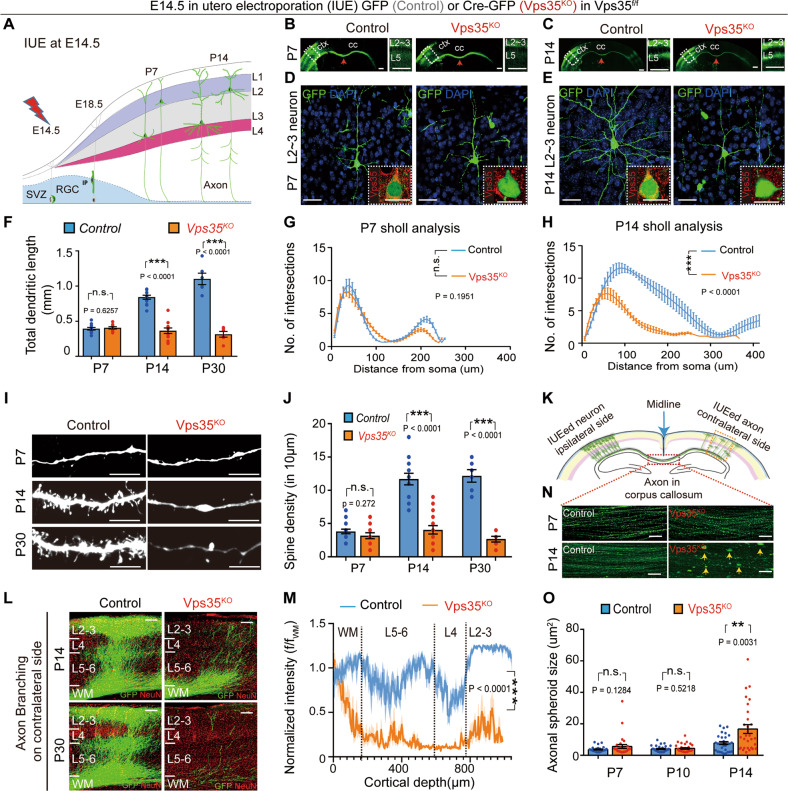
Defective dendritic and axonal morphogenesis in neonatal pyramidal neurons by depletion of Vps35 at E14.5 NSCs/NPCs. Vps35^f/f^ embryos were in utero electroporated (IUEed) with plasmids of GFP (control) or Cre-GFP (Vps35^KO^) at E14.5 and their neocortical brain sections were examined at indicated ages. **a** Schematics of E14.5 IUE experimental procedures. **b**, **c** Representative images from neocortical brain sections at indicated ages. Cerebral cortex (ctx), corpus callosum (cc), and middle lines (red arrows) are marked. Images in dashed lines are amplified in the right panels. Cortical layers (L) 2–3 or 5 are indicated. **d**, **e** Representative Z-stack projection images of GFP^+^ (electroporated) L2–3 cortical neurons. The inserts are representative images of immunostaining analysis with anti-Vps35 (red). **f** Quantification analysis of the cumulative length of the dendritic processes of L2–3 neurons. (*n* = 12 neurons from four mice per group, unpaired two-tailed *t*-test). **g**, **h** Sholl analyses of dendritic intersections from neuronal soma. (*n* = 4 neurons from three mice per group; two-way ANOVA with a Tukey’s multiple comparisons test). **i** Examples of reconstructed dendritic branch segments and their associated spines at indicated ages. **j** Quantification analysis of the spine density. (*n* = 16 neurons from four mice per group, unpaired two-tailed *t*-test). **k** Diagram showing IUEed axons in corpus callosum (CC) crossing the midline and then innervation into contralateral cortex and branching extensively at in layers 2–3 and layer 5. **l** Higher magnification of contralateral side showing reduced axon innervation and branching in Vps35-KO neurons. **m** Quantification of fluorescence intensity across layers normalized by fluorescence of the underlying white matter (f/f_wm_) from P14 sections. **n** Representative images of GFP^+^ axon taken from the illustrated area in **k**. The yellow arrows indicate the axonal spheroids. **o** Quantification analysis of data in **n**. (*n* = 30 from six mice per group, unpaired two-tailed *t*-test). Scale bars: in **b**, **c** and **l**, 500 μm; and in **d**, **e**, **i**, and **n**, 10 μm. Individual data points were shown as dots with group mean ± s.e.m; **P < 0.01, ***P < 0.001; n.s. not significant.

Studying in vivo function of Vps35 has been difficult because *Vps35* null allele die before E9.5 with an unknown reason [10]. To determine Vps35’s function during neocortical development, we performed IUE to delete *Vps35* in a subset of neocortical pyramidal neurons. Cre-encoding plasmids were electroporated into the neural stem cells (NSCs) or neural progenitor cells (NPCs) in the ventricular zone of *Vps35*^*f/f*^ embryos at E14.5. These NSCs/NPCs differentiate into neocortical pyramidal neurons at layer (L) 2–3 at neonatal age (Fig. 1a) [40]. As expected, expression of the Cre-GFP abolished endogenous Vps35’s expression (Fig. 1d inserts). At P7, most of *Vps35*^*KO*^ (Cre-GFP^+^) neurons had migrated to neocortical L2–3, as that of control (GFP^+^) neurons (Fig. 1b). Their dendritic morphology (Fig. 1d), length (Fig. 1f), complexity (Fig. 1g), and their axonal length and distribution in corpus callosum (CC) (Fig. 1n) appeared to be comparable with those in control neurons. These results suggest little impact if there is any of *Vps35*^*KO*^ on neuron migration and axonal initiation.

However, starting from P14, obvious morphological changes in both dendrites and axons were found in *Vps35*^*KO*^ neurons (Fig. 1e). Quantification of the total dendritic length indicated a progressive reduction of dendritic processes in *Vps35*^*KO*^ neurons (Fig. 1f). Sholl analysis, which quantifies the complexity of the dendritic arbor, showed much lower number of dendritic intersections in *Vps35*^*KO*^ neurons (Fig. 1h). These results suggest that *Vps35*^*KO*^ prevents dendritic arborization during neuronal maturation. Dendritic arborization and spine formation are closely related, we then examined spine morphology and quantified spine density. By 3D reconstruction, a significant reduction in total spine density was detected in *Vps35*^*KO*^ neurons at P14 and P30 (Fig. 1i–j).

As IUE was performed unilaterally in the somatosensory cortex, and the axon of electroporated neurons was determined by expression of GFP, which allowed us to check the axonal morphology at high resolution. As shown previously [22], callosal axons cross the cortical midline, forming the CC around birth, and then reach the contralateral cortex during the first postnatal week and branch extensively at P14 which targeting the contralateral layers 2–3 and layer 5 neurons (Fig. 1k). *Vps35*^*KO*^ axons formed, grew, and successfully crossed the midline (Fig. 1b, c), but failed to branch and innervation contralateral side neurons at P14. These deficits seemed to be more severe at P30 (Fig. 1l and m). In addition, a marked increase of axonal spheroid formation (viewed by GFP with area size >10 µm^2^) was observed in *Vps35*^*KO*^ axons in CC (Fig. 1n and o).

To further determine neuronal Vps35’s function, we generated *Vps35*^*f/f*^*; Neurod6-Cre* (referred to hereafter as *Vps35*^*Neurod6*^) mice by crossing *Vps35*^*f/f*^ with *Neurod6-Cre* (also called *Nex-Cre*). The *Neurod6-Cre* is expressed exclusively in pyramidal neurons beginning at approximately E11 [21]. *Vps35*^*Neurod6*^ mice exhibited no obvious differences from control littermates at birth (Fig. S1A). However, they lagged in postnatal development including cessation of weight gain after 1 week and neonatal lethality before P23 (Fig. S1A, B). Consistent with *Neurod6-Cre*, Vps35 was depleted in differentiated pyramidal neurons in neocortex by immunostaining analysis (Fig. S1C). Western blot analysis also showed selective reductions of Vps35 protein levels in *Vps35*^*Neurod6*^ neocortex and hippocampus (59.3 ± 6.29% reduction in neocortex and 56.61 ± 3.19% in hippocampus), but not in other brain regions where the Cre is not expressed (Fig. S1D).

**Fig. 2:**
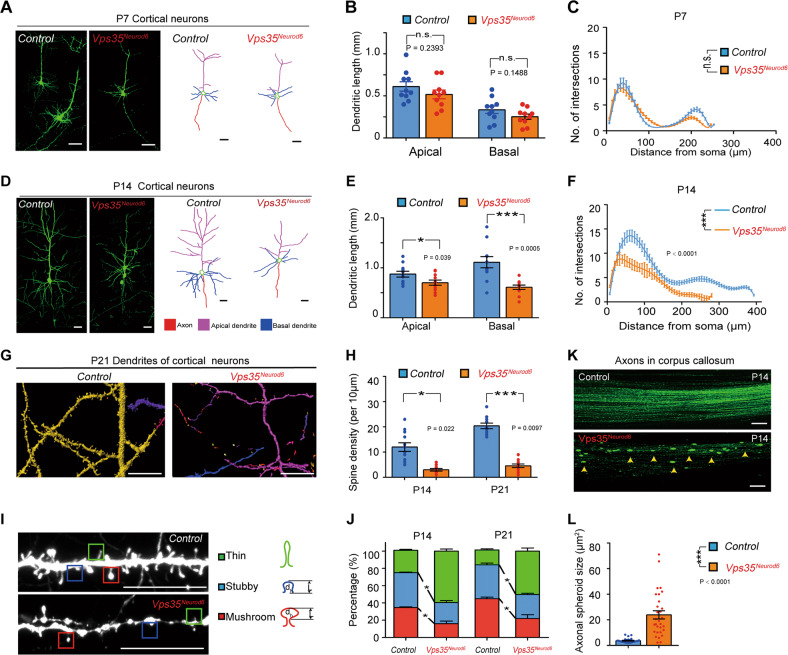
Dendritic maturation defect and axonal spheroid formation in Vps35^Neurod6^ neocortex. **a**, **d** Representative images of EYFP^+^ cortical neurons (left) and reconstructed neurons (right) at P7 and P14. **b**, **e** Quantification analysis of the apical and basal dendritic length. (*n* = 12 neurons from four animals per genotype; unpaired two-tailed *t*-test). **c**, **f** Sholl analysis for dendritic complexity. (two-way ANOVA with a Tukey’s multiple comparisons test). **g** Representative 3D-reconstructed dendritic spines from P21 brain sections. **h** Quantifications of spine density at ages of P14 and P21. (*n* = 12 from four mice per group, unpaired two-tailed *t*-test). **i** Representative Z-stack projection images of dendritic spines. Spines are categorized as mushroom (red), stubby (blue), and thin (green) subtypes. **j** Quantification analysis of data in **h** for percentage of different types of spines of neurons at ages of P14 and P21. (Four neurons from four mice per group, two-way ANOVA with Tukey’s multiple comparison test). **k** Confocal images of GFP^+^ projecting axons in CC after IUE of CAG-GFP at E14.5 control (Vps35^f/f^) and Vps35^Neurod6^ embryos. **l** Quantification analysis of data in **k**. (*n* = 30 from three mice per group, unpaired two-tailed *t*-test). Individual data points were shown as dots with group mean ± s.e.m. *P < 0.05; ***P < 0.001; n.s. not significant.

To better view the morphology of individual pyramidal neurons in *Vps35*^*Neurod6*^ mice, we crossed *Vps35*^*Neurod6*^ with a *Thy1-EYFP* mice, which fluorescently labels a proportion of neurons in neocortex. The EYFP expressing dendrites from *Vps35*^*Neurod6*^ mice was comparable with control mice at P7 (Fig. 2a–c). However, at P14, these *Vps35*^*Neurod6*^ neurons showed similar deficits as that detected by IUE assay, which include reduced dendritic length (Fig. 2d, e) and complexity (Fig. 2f), decreased dendritic spine density (Fig. 2g, h), and impaired mature spine formation (Fig. 2i, j). Moreover, *Vps35*^*Neurod6*^ axons in CC also displayed increased axonal spheroids (Fig. 2k–l). The dendritic phenotypes were further confirmed by Golgi staining analysis of P14 control and *Vps35*^*Neurod6*^ cortical brains. Not only L5 neurons, nearly all neurons marked by Golgi staining showed abnormal morphology in the mutant neocortex, including reduced dendritic length and complexity and decreased dendritic spine density (Fig. S2). These results suggest a function of Vps35 in promoting dendritic and axonal maturation in developing pyramidal neurons.

### Concurrence of neurodegenerative and reactive glial responses in *Vps35*^*Neurod6*^ neocortex

**Fig. 3:**
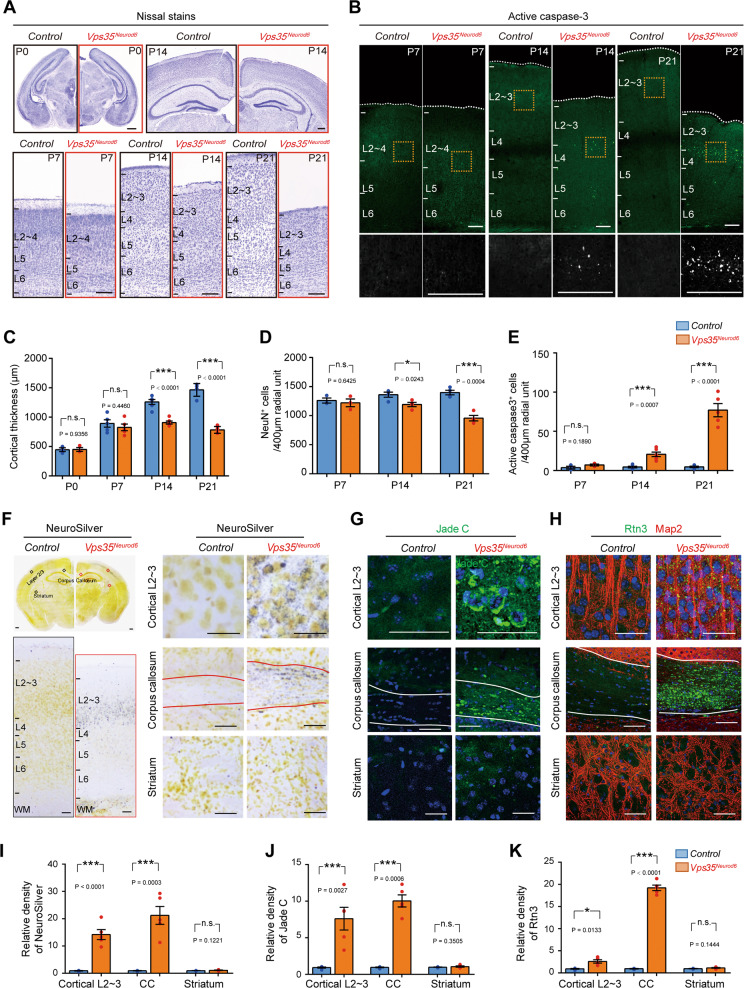
Degenerative cortical neurons in *Vps35*^*Neurod6*^ brain. **a** Representative Nissl stains of control (*Vps35*^*f/f*^) and *Vps35*^*Neurod6*^ animals at indicated ages. **b** Representative images of active caspase-3 immunostaining analysis of neocortex at indicated ages. **c** Quantification analysis of Nissl stains that revealed a significant reduction in cortical thickness of *Vps35*^*Neurod6*^ animals at P14/P21, but not at P0/P7, as compared with those of control mice (*n* = 3–4 animals per genotype; two-tailed unpaired *t*-test). **d** Quantification analysis of NeuN^+^ neuron numbers in the neocortex that revealed no significant change at P7, but losses of 12% and 29% of cortical neurons at P14 and P21, respectively (*n* = 3–4 animals per genotype; two-tailed unpaired *t*-test). **e** Quantification analysis of active caspase-3^+^ cells that showed an age-dependent increase in apoptotic cells in *Vps35*^*Neurod6*^ neocortex at indicated ages (*n* = 3–4 animals per genotype; two-tailed unpaired *t*-test). **f** Images of NeuroSilver-stained brain sections from control and *Vps35*^*Neurod6*^ mice. Positive signals (dark gray/black staining) were detected in cortical L2–3 neurons and corpus callosum [major white matter (WM) axonal pathways], but not in striatum. Representative images of Jade C staining (**g**) and anti-Rtn3 immunostaining (**h**) in cortical L2–3, corpus callosum, and striatum of Control and *Vps35*^*Neurod6*^ mice. **i**–**k** Quantification analysis of data from (**f**–**h**). (*n* = 4 animals per genotype; two-tailed unpaired *t*-test). Scale bars: in **a**, **b**, and **f**, 100 μm; and in **g** and **h**, 20 μm. Individual data points were shown as dots with group mean ± s.e.m; *P < 0.05; ***P < 0.001; n.s. not significant.

The morphological changes in neonatal *Vps35*^*Neurod6*^ neurons resemble in certain degree to the neurodegenerative pathology for aged neurons [41]. We thus examined if *Vps35*^*Neurod6*^ neocortex shows any feature of neurodegeneration. By analysis of Nissl-stained brain sections, we did not observe any change at P0 and P7 but noticed an obvious reduction in cortical thickness in *Vps35*^*Neurod6*^ at P14 that was more severe at P21 (Fig. 3a and c). NeuN (a marker for neurons) immunostaining analysis showed a comparable number of cortical neurons at P7 with those of control mice, but losses of 12% and 29% of cortical neurons at P14 and P21, respectively (Fig. 3d). Such an age-dependent neuron loss was accompanied by an increase in active caspase-3 positive apoptotic cells in *Vps35*^*Neurod6*^ neocortex (Fig. 3b and e). In line with the enhanced active caspase-3 staining, the TUNEL assay confirmed an increase of apoptotic cells in P21 *Vps35*^*Neurod6*^ neocortex (Fig. S3A, B). As apoptosis can be induced by various cellular stresses, including DNA damage, the P21 control and *Vps35*^*Neurod6*^ brain sections were immune-stained with antibodies against γH2AX and P53, both DNA damage markers [42, 43]. Indeed, both γH2AX and P53 were increased in *Vps35*^*Neurod6*^ neocortex as compared with that of littermate controls (Figs. S3B–C). These results suggest that the DNA damage associated apoptosis may contribute to the progressive cortical neuron loss in *Vps35*^*Neurod6*^ mice.

Notice that the percentage apoptotic cells (less than 1%) in *Vps35*^*Neurod6*^ neocortex were smaller than that of neuron loss, suggesting additional mechanism(s) underlying cortical brain atrophy. We thus further examined neurodegenerative pathology in *Vps35*^*Neurod6*^ brain sections by staining with NeuroSilver (Fig. 3f) or Jade C (Fig. 3g), or immunostaining using anti-Rtn3 (Fig. 3h), as these three markers are frequently used for detection of neuronal damage or dystrophic neuritis [28, 29, 44]. Indeed, positive signals with NeuroSilver, Jade C, and Rtn3 were all detected in *Vps35*^*Neurod6*^, but not control brains (Fig. 3f–k). Interestingly, staining signals of the three markers were stronger in the mutant L2–3 and CC axon regions (Fig. 3f–k), suggesting a regional difference/selectivity of neurodegeneration. Notice that in the mutant stratum, where the *Neurod6-Cre* is not expressed, the staining signals appeared to be comparable with that in controls (Fig. 3f–k), demonstrating the specificity of the staining.

**Fig. 4:**
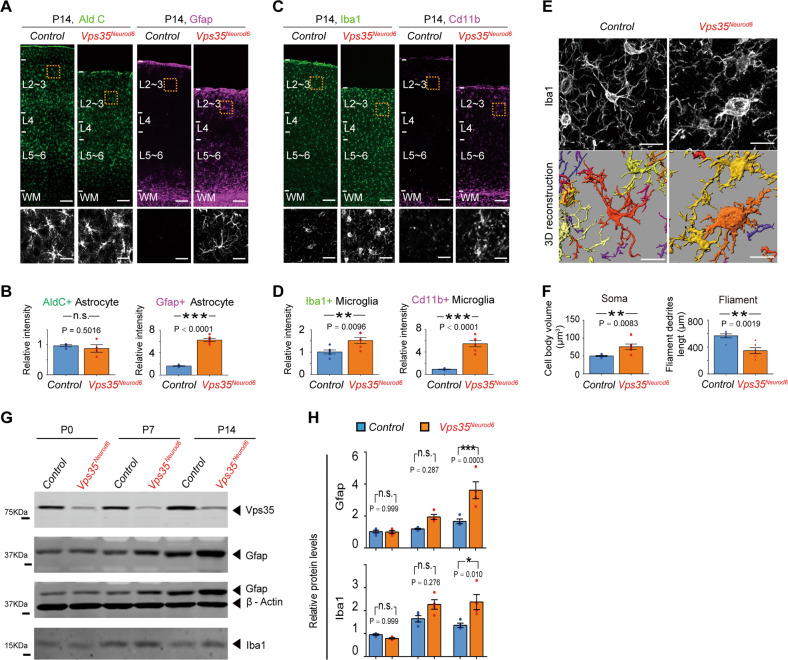
Reactive astrocytes and microglia in Vps35^Neurod6^ neocortex. **a**, **c** Immunostaining analysis using indicated antibodies in P14 neocortical sections. Higher-magnification images of the boxed regions were shown in lower panels. **b, d** Quantification of immunofluorescence from **a** and **c**. (*n* = 6 animals per genotype; two-tailed unpaired *t*-test). **e, f** Representative 3D reconstruction of cortical microglial morphology and Imaris-based automatic quantification of microglial morphology. (*n* = 6 cells from three mice per genotype; two-tailed unpaired *t*-test). **g**, **h** Western blot analyses and quantification analysis, which confirmed increased Gfap and Iba1 protein levels in the *Vps35*^*Neurod6*^ forebrains at P14, but not P0 and P7. (*n* = 4 animals per genotype; one-way ANOVA with a Tukey’s multiple comparisons test). Scale bars: in **a**, **c**, and **i** (upper panel), 100 μm; and in **a**, **c**, **i** (lower panel) and **e**, 5 μm. Individual data points were shown as dots with group mean ± s.e.m; *P < 0.05; **P < 0.01; ***P < 0.001; n.s. not significant.

Neurodegeneration is often associated with glial activation. As shown in Fig. 4a, whereas aldolase C positive (^+^) astrocytes in P14 *Vps35*^*Neurod6*^ neonatal cortex was comparable with those of control brains, Gfap^+^ astrocytes were significantly elevated in the mutant neocortex, suggesting an increase of reactive astrogliosis, but not astrogliogenesis or astrocyte differentiation (Fig. 4a, b). In accord, Iba1^+^ as well as Cd11b^+^ microglia were both increased in the mutant neocortex (Fig. 4c, d), indicating an elevation of microglial activation. This view was in line with the observation of altered Iba1^+^ microglial morphology in *Vps35*^*Neurod6*^ neocortex, which had larger cell body and shorter filopodium-like processes than those in the controls (Fig. 4e, f), exhibiting morphological features of microglial activation. The increased Gfap and Iba1 protein levels in *Vps35*^*Neurod6*^ cortical homogenates (from P14, but not P0 and P7, brains) was also detected by western blot analyses (Fig. 4g, h), providing additional support for elevated reactive astrogliosis and microglial activation.

### Increased autophagosome and neurodegeneration associated proteins in *Vps35*^*Neurod6*^ brains

**Fig. 5:**
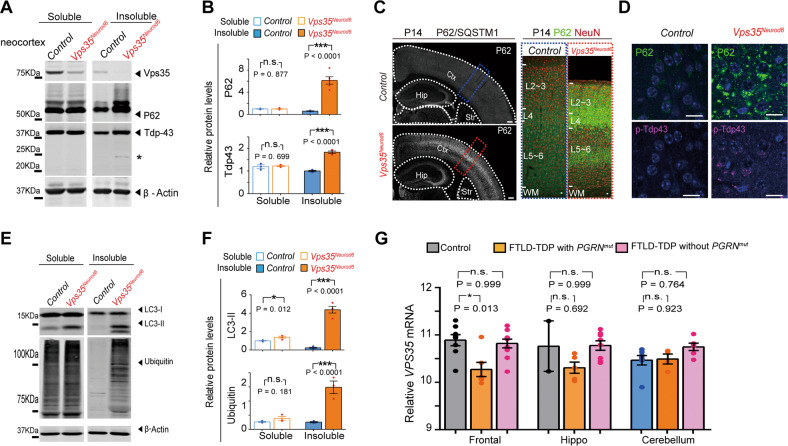
FTD-like neuropathology in *Vps35*^*Neurod6*^ neocortex. **a** and **e** Western blot analyses of soluble (by 1% Triton X-100) and insoluble fractions (by 7 M Urea, 2 M Thiourea, 4% CHAPS) of homogenates of P14 control and *Vps35*^*Neurod6*^ neocortex using indicated antibodies. **b**, **f** Quantification analysis of relative protein expression levels from **a** and **g**. (*n* = 4 animals per genotype; two-tailed unpaired *t*-test). **c** Tile scan assembly of a coronal section of P14 brain immunostained with anti-P62. Ctx: Cortex; Hip hippocampus and Str Striatum. **d** Representative images of co-immunostaining analysis with antibodies against P62 (green) and p-Tdp43 (purple) of P14 cortical sections. **g** Analysis of *VPS35* mRNA levels in the frontal lobe of cortex (frontal), hippocampus (hippo), and cerebellum from a cohort of eight unaffected (Control), six FTD-TDP patients with Pgrn mutation, and ten FTD-TDP without Pgrn mutation. (Data were from GEO GSE20141). Expression levels are normalized to mean of the unaffected group (One-way ANOVA with Tukey’s multiple comparison test). Scale bars: in **c**, 500 μm; in **d**, 10 μm. Individual data points were shown as dots with group mean ± s.e.m; *P < 0.05, **P < 0.01, ***P < 0.001; n.s. not significant.

To further address whether *Vps35*^*Neurod6*^ neocortex show neurodegeneration-like pathology, we specifically examined FTD associated proteins, as FTD is the 2nd most common younger-onset dementia that is due in large to the neurodegeneration in pyramidal neurons of frontal lobe [45]. In most of FTD cases, neocortical pyramidal neurons contained pathological protein aggregation of TDP43 and P62 [46]. Western blot analysis showed increased P62 and Tdp43 levels in insoluble fractions of homogenates of P14 *Vps35*^*Neurod6*^ cortex (Fig. 5a, b). In addition, a 20–25 kDa band (likely to be a cleaved C-terminal fragment of *Tdp43*) was detected in *Vps35*^*Neurod6*^ insoluble fractions (Fig. 5a, asterisk). Immunostaining analysis showed a more dramatic increase of P62 in the neocortex of P14 *Vps35*^*Neurod6*^ cortex (Fig. 5c, d). Intracellular aggregates of phosphorylated *Tdp43* was also noted in *Vps35*^*Neurod6*^ cortical neurons (Fig. 5d). Furthermore, both ubiquitin-conjugated proteins (viewed by anti-ubiquitin) and LC3-II (a marker of autophagosomes) were markedly increased in the insoluble fractions of homogenates from *Vps35*^*Neurod6*^ neocortex (Fig. 5e, f). These results provide additional support for Vps35-KO pyramidal neurons to undergo neurodegeneration with characteristics of FTD.

We further analyzed *Vps35*’s mRNAs in control and different groups of human patients with FTD-TDP. Interestingly, *Vps35*’s expression was selectively reduced in the frontal lobe of patients with FTD-TDP carrying *Progranulin* (*PGRN*) mutations, but not in FTD-TDP patients without *PGRN* mutation, as compared with unaffected controls (Fig. 5g). These results support the view for Vps35-deficiency as a potential risk factor for FTD pathogenesis.

### Increase of Sort1 in lysosomes of *Vps35*-KO neurons

The selective reduction of *Vps35*’s mRNA in the frontal lobe of FTD patients with *PGRN* mutation leads us to wonder if Pgrn is involved in *Vps35-KO*-induced FTD-like brain deficits. Pgrn-deficiency is believed to be a risk factor for FTD [47], and it is also implicated in enhancing neurite outgrowth and neural survival in primary cultured neurons [48], and in regulating neuronal lysosomal function [49, 50]. Interestingly, the Pgrn levels were reduced in *Vps35*^*Neurod6*^ neocortex (Fig. S4A, B). We thus asked if the decreased Pgrn in *Vps35*^*Neurod6*^ brain contributes to the *Vps35*-KO-induced neuropathology. To this end, we generated Pgrn expression plasmid and co-electroporated it with Cre into E14.5 *Vps35*^*f/f*^ embryos, and asked whether Pgrn expression in *Vps35*-KO neurons could diminish the dendritic and axonal deficits. To our surprise, no significant difference, in viewing dendritic and axonal phenotypes, was detected in *Vps35*-KO (*Cre*-GFP^+^) neurons with or without Pgrn expression (Fig. S4C–G). These results suggest that expression of Pgrn in *Vps35*-KO neurons is insufficient to rescue *Vps35*-KO-induced neuronal deficits.

We then examined Sort1, as it not only acts as a receptor for Pgrn [19] but also a cargo of Vps35 in Hela cells and COS-7 Cells [51, 52]. Whereas Pgrn was reduced, Sort1 levels were slightly increased in P1 *Vps35*^*Neurod6*^ neocortex (Fig. S5A, B). *Sort1*’s mRNAs were comparable in *Vps35*^*Neurod6*^ neocortex with that of controls (Fig. S5C). We then examined Sort1 subcellular distribution in *Vps35*-KO neurons. Elevated Sort1 in both Rab5^+^ endosomes and Lamp1^+^ late endosomes/early lysosomes were detected in primary cultured *Vps35*-KO neurons (Fig. S5D–E). Such an altered Sort1 distribution (increase in Lamp1^+^ lysosomes, but decrease in GM130^+^ Golgi) was also detected in neocortical neurons of *Vps35*^*Neurod6*^ brain (Fig. S5F, G). Furthermore, western blot analyses of lysates of purified lysosomal and surface fractions showed an increase of lysosomal Sort1, but a decrease of cell surface Sort1 in *Vps35*^*Neurod6*^ mice (Fig. S5H, I), reconfirmed the observation of increased lysosomal Sort1 in the mutant neurons. These results thus support the view for Sort1 as a cargo of Vps35 in developing pyramidal neurons and implicate a delayed or impaired lysosomal degradation of Sort1 in Vps35-KO neurons.

### Sort1 contributing in part to *Vps35-KO*-induced neurodegenerative pathology

**Fig. 6:**
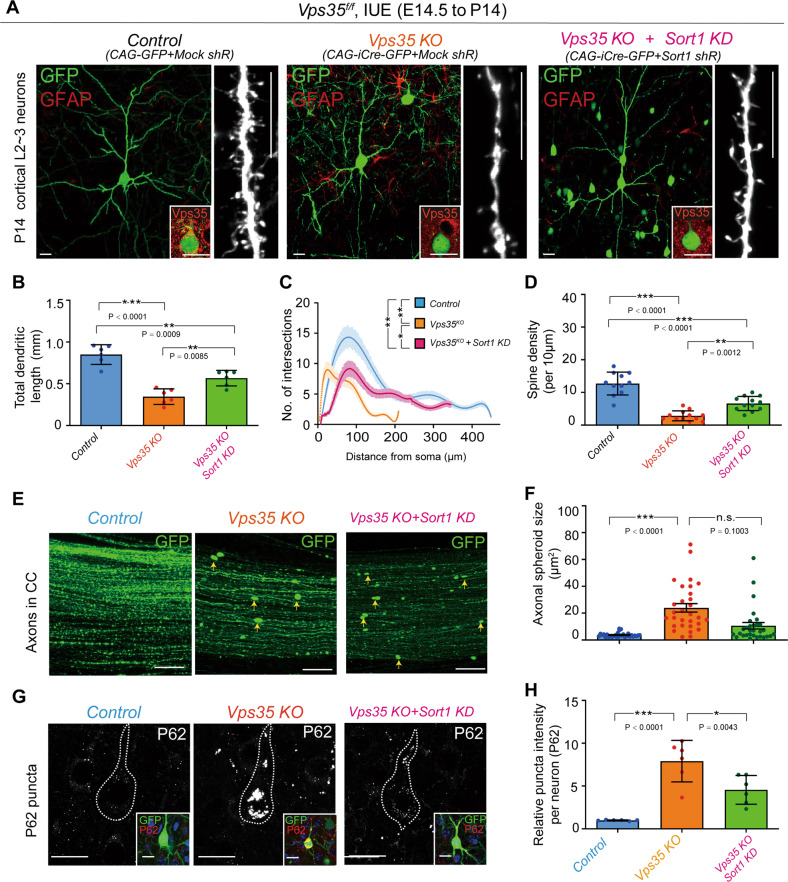
Attenuated dendritic deficits in *Vps35*-KO neurons by suppression of Sort1 expression. **a** Representative images of P14 cortical L2–3 neurons which were electroporated at E14.5 into *Vps35*^*f/f*^ embryos with the indicated plasmids. The inserts are representative images of immunostaining analysis with anti-Vps35. Quantification analysis of total dendritic length (**b**), dendritic complexity (**c**), and spine density (**d**). *n* = 6 neurons from three mice per group; one-way ANOVA with Tukey’s multiple comparison test). **e** Representative images of GFP^+^ projecting axons in CC taken from indicated P14 neocortical brains. **f** Quantification analysis of data in **h** (*n* = 30 from three mice per group, one-way ANOVA with Tukey’s multiple comparison test). **g** Representative images of immunostaining analysis using anti-P62 of cortical L2–3 neurons from electroporated brain with indicated constructs. Inserts indicated which neurons were transfected. **h** Quantification analysis of data in **g**. (*n* = 6 neurons from three mice per group, one-way ANOVA with Tukey’s multiple comparison test). Scale bars: in **a**, **e**, and **g**, 10 μm. Individual data points were shown as dots with group mean ± s.e.m; *P < 0.05, **P < 0.01, ***P < 0.001; n.s. not significant.

To further investigate Sort1’s contribution to *Vps35-KO*-induced deficits, we examined if the increased Sort1 in *Vps35*^*Neurod6*^ brain is necessary for *Vps35*-KO-induced neuropathology. The plasmids encoding shRNA-*Sort1* (#2 and #3) were generated, but the shR-*Sort1 (*#3) efficiently suppressed the expression of Sort1 in vitro and in vivo (Fig. S6A, B). The shR-*Sort1(#3)* plasmids were co-electroporated with *Cre*-GFP into *Vps35*^*f/f*^ mouse embryos. Interestingly, dendritic deficits (including reduced length and complexity and decreased spine density) were diminished when shR-*Sort1*(#3) was co-expressed (Fig. 6a–d). However, the axonal spheroid formation remained unchanged (Fig. 6e, f). Notice that the increased P62 in *Vps35*-KO neurons was attenuated by co-electroporation of shR-*Sort1*(#3) (Fig. 6g, h). In addition, the shR-*Sort1(#3)* plasmids were electroporated into *Vps35*^*Neurod6*^ embryos (at E14.5), and their P14 dendritic deficits in *Vps35*^*Neurod6*^ neocortex were also diminished by suppression of Sort1 expression (Fig. S6C–G). Together, these results suggest a partial rescue of *Vps35*-KO-induced neuronal deficit by suppression of Sort1’s expression.

### Accumulation of lysosomal Sort1 causing similar dendritic and axonal deficits as that of Vps35-KO

**Fig. 7:**
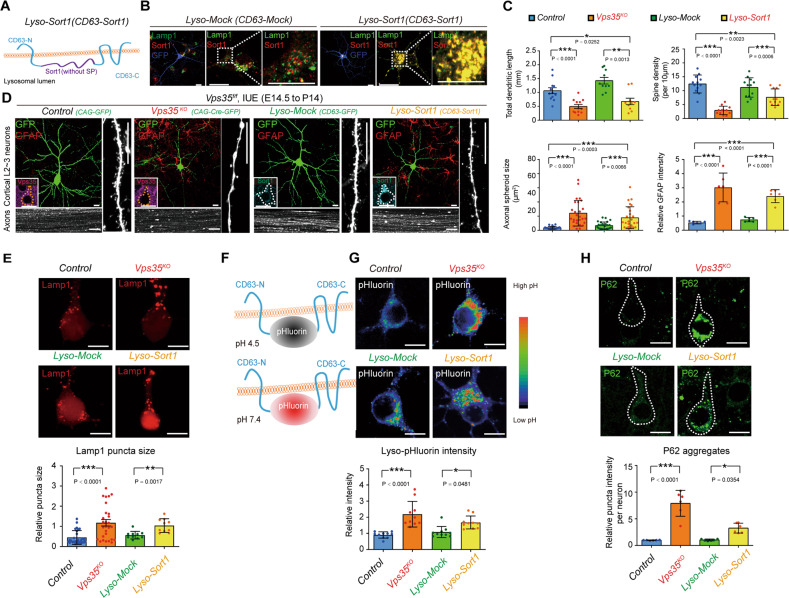
Impairment in axonal and dendritic terminal differentiation and impaired lysosomal acidification in neurons expressing *Lyso-Sort1* fusion protein. **a** Schematic of the *Lyso-Sort1* construct. **b** Confocal images of a DIV 10 neuron expressing *CD63-Mock (Lyso-Mock)* and *CD63-Sort1 (Lyso-Sort1)*, which were co-immunostained with anti-Lamp1 (green) and Sort1 (red). **c** Quantification analysis of total dendritic length, spine density, axonal spheroid size, and relative GFAP intensity from (**f**). (*n* = 12 neurons from three mice per group; one-way ANOVA with Tukey’s multiple comparison test). **d** Representative images of P14 cortical L2–3 neurons and their axons in CC which were electroporated at E14.5 with indicated plasmids. The inserts are representative images of immunostaining analysis with anti-Vps35 (purple) or anti-Sort1 (cyan) in the electroporated neurons. **e** Representative images and quantitation analysis of Lamp1^+^ puncta size in indicated group. Quantification analysis showed increased Lamp1 puncta size in *Vps35*^*KO*^ and *Lyso-Sort1* expressed neurons. (*n* = 10 neurons per group; two-tailed unpaired *t*-test). **f** A lysosomal target pH-sensitive optical reporter (*CD63-pHluorin* or *Lyso-pHluorin*) is quenched when exposed in the acidic environment. When pH is neutralized, CD63-pHluorin increase the fluorescent intensity. **g** Representative images of neurons co-transfected with *Lyso-pHluorin*. Quantification analysis showed increased intra-lysosomal pH in *Vps35*^*KO*^ and *Lyso-Sort1* expressed neurons. (*n* = 10 neurons per group; two-tailed unpaired *t*-test). **h** Representative images of immunostaining analysis of P62 in cortical neurons. Quantification analysis showed increased P62 aggregates in *Vps35*^*KO*^ and *Lyso-Sort1* expressed neurons and indicative of defects in the clearance of autophagosomes. (*n* = 6 neurons from three mice per group, one-way ANOVA with Tukey’s multiple comparison test). Scale bars: in **b**, **d**, **e**, **g**, and **h**, 10 μm. Individual data points were shown as dots with group mean ± s.e.m; *P < 0.05, **P < 0.01, ***P < 0.001; n.s. not significant.

We next wondered if Sort1-increase in lysosomes plays a role in *Vps35*-KO-induced dendritic and axonal defects. To this end, a plasmid encoding Lyso-*Sort1* fusion protein was generated, in which, *Sort1* was fused with *CD63*, a lysosome membrane protein, and thus targeting to lysosomes efficiently and specifically [53] (Fig. 7a, b). Interestingly, when this *Lyso-Sort1* fusion plasmid was IUEed into E14.5 embryos, the resulting P14 neocortical neurons exhibited similar deficits as that of *Vps35*-KO neurons, which include impaired dendritic growth, reduced spine density, and axonal spheroid formation (Fig. 7c, d). GFAP^+^ astrocytes surround with *Lyso-Sort1* neurons too (Fig. 7d). In contrast, neurons expressing *Lyso-Mock (CD63)* appeared to be normal, suggesting the specificity of *Lyso-Sort1* fusion protein’s effect. These results suggest that selective expression of Sort1 in lysosomes is sufficient to recapitulate *Vps35*-loss-induced terminal differentiation defects.

### Lysosomal deficits in both Vps35-KO and Lyso-Sort1 expressing neurons

The similar deficits between Vps35-KO and Lyso-Sort1 expressing neurons led to the speculation that both Vps35-KO and Lyso-Sort1 expression may cause similar lysosomal defect. Notice that the *Lamp1*^+^ lysosomal size was enlarged in both *Vps35*-KO and Lyso-Sort1 expressing neurons (Fig. 7e), suggesting an alteration of lysosomal morphology. We then examined lysosomal pH or acidification, an essential event for lysosomal function [54]. The intra-lysosomal pH was measured by Lyso-pHluorin, which shows strong green fluorescence when it is exposed to a basic or deacidified environment [53] (Fig. 7f). Interestingly, increased Lyso-pHluorin fluorescence intensities were detected in both *Vps35*-KO and Lyso-Sort1 expression neurons (Fig. 7g), suggesting an impaired lysosomal acidification in both *Vps35*-KO and Lyso-Sort1 expression neurons. This view was also supported by the observations of increased autophagic protein, P62, in both *Vps35*-KO and Lyso-Sort1 expression neurons (Fig. 7h). Together, these results suggest that both Vps35-KO and Lyso-Sort1 may cause dendritic and axonal deficits and neurodegenerative pathology in developing neurons likely by impairing lysosomal acidification.

## Discussion

Vps35 dysfunction is considered to be a risk factor for neurodegenerative diseases, including AD and PD [55]. In this paper, we provide evidence that *Vps35*-deficiency in developing neurons not only impairs axon and dendrite terminal differentiation, but also causes neurodegenerative pathology. Loss of Vps35 in cortical pyramidal neurons results in dendritic morphogenesis and maturation defects and axonal spheroid formation (Figs. 1 and 2). *Vps35*-cKO mice, *Vps35*^*Neurod6*^, show early onset cortical brain degenerative pathology, including progressive cortical atrophy and increase of neuronal death, “degenerative neuronal morphology” marked by NeuroSilver, Jade C, and Rtn3 (Fig. 3), reactive glial responses labeled by Gfap^+^ astrocytes and Cd11b^+^ microglia (Fig. 4). In addition, *Vps35*^*Neurod6*^ neocortical brains display accumulations of P62, Tdp43, phospho-Tdp43, ubiquitin-conjugated proteins, and LC3-II proteins, exhibiting features of FTD-like neuropathology (Fig. 5). Lysosomes are enlarged with acidification defects in Vps35-KO neurons (Fig. 7). We also uncovered Sort1 as a key cargo of Vps35, whose accumulation in lysosomes contributes to Vps35-KO-induced neuronal deficits (Figs. 6 and 7). Our results not only demonstrate a critical role of Vps35 in neuronal terminal differentiation, but also uncover a link of defective terminal differentiation of neurons with neurodegeneration, reveal the importance of lysosomal function in both processes, and implicate Vps35-deficiency as a potential risk factor for FTD development. These findings add further insights into Vps35’s function and its involvement in neurodegenerative diseases.

Lysosome and autophagy dysfunctions are known to be key players in the pathogenesis of neurodegenerative diseases [56, 57]. Here, we speculate that normal lysosomal function may also be required for terminal axonal and dendritic differentiation in developing neurons, because lysosomal dysfunction induced by Vps35-loss or expression of Lyso-Sort1 fusion results in similar deficits in axonal and dendritic terminal differentiation, in addition to neurodegenerative pathology (see Fig. 7). How does Vps35 regulate lysosomal and autophagy functions? Vps35-deficiency in Hela cells reduces proper delivery of proteases to the lysosomes through CI-M6PR; [58, 59] Vps35-deficiency also impairs autophagic degradation in *Drosophila* [60] and autophagosome formation in Hela cells (through Atg9a);[61] and Vps35-loss increases the degradation of Lamp2a in mouse dopaminergic neurons leading to a decreased chaperone-mediated autophagy [14]. Here, we found that Vps35 regulates lysosomal acidification/maturation in developing pyramidal neurons. While Lamp1^+^ early lysosomes are enlarged in *Vps35*-KO neurons, the acidic lysosomes (or mature lysosomes) are reduced (Fig. 7e–g). In line with this view are our observations of increased *P62* and *LC3-II* autophagic proteins in *Vps35*^*Neurod6*^ pyramidal neurons (Figs. 5 and 7h). Based on our results, we speculate that Sort1, a cargo of Vps35, may be also involved in Vps35 regulation of lysosomal acidification/functions. Intestinally, Sort1 is identified as a binding partner of ATP6AP2, a critical protein for v-ATPase and lysosomal acidification, by proteomics-based approach [62]. Thus, we posit that Vps35-KO in developing pyramidal neurons increases lysosomal Sort1, which may inhibit ATP6AP2 regulated v-ATPase activity and lysosomal acidification. Whereas these observations support the view depicted in Fig. S7, we cannot exclude the possible involvement of other retromer cargos, such as CI-M6PR [58, 59] and Lamp2a [14], which may also underlie Vps35 regulation of lysosomal functions.

Besides lysosome and autophagy dysfunctions, deregulation of apoptosis is associated with neurodegenerative disorders [63, 64]. Here we showed that ablation of Vps35 in developing pyramidal neurons resulted in not only accumulations of LC3^+^ and P62^+^ autophagosome markers and FTD-like neurodegenerative pathology, but also an increase of caspase-3-induced apoptosis. Interestingly, both apoptotic neurons [65] and P62^+^ inclusions [66] are also found in the cortex of FTD patients. While we believe that the dysfunctional lysosomes contribute to the increase of autophagosome and neurodegeneration associated proteins in *Vps35*^*Neurod6*^ cortex, the mechanisms underlying the apoptosis remain largely unclear. We speculate the following possibilities. First, Vps35-deficiency may induce DNA damage associated apoptosis by impairing mitochondrial fusion and function and increasing reactive oxygen species (ROS). This view is in line with previous publications that Vps35-deficiency in DA neurons causes neuronal loss by impairing mitochondrial fusion and function [12, 13]. and ROS increases DNA damage [67]. In addition to DA neurons, impaired mitochondrial fusion was also detected in Vps35-deficient pyramidal neurons (data not shown). Whereas these observations support the view for a mitochondrial dysfunction to underlie Vps35-deficiency induced apoptosis, further experiments are necessary to determine if ROS is increased and contributes to the DNA damage and apoptosis in Vps35 mutant cortex. Second, it is of interest to note a recent study [68] that shows Vps35 to be a novel regulator of antiapoptotic protein Bcl-xL; and Vps35-depleted cells display an enhanced rate of apoptosis by facilitating Bcl-xL’s transport to the mitochondrial outer membrane. Thus, further investigating Vps35’s function in regulating Bcl-xL trafficking in cortical neurons and Bcl-xL’s involvement in the apoptosis induction in Vps35-deficient neurons are necessary. Third, it is noteworthy that Vps35/retromer is implicated in apoptotic cell clearance through sorting of CED-1, a phagocytic receptor for apoptotic cells identified in Caenorhabditis elegans [69]. Thus, it will be of interest to address if loss of Vps35/retromer in neurons increases apoptotic cells due to an impairment in their clearance by microglial cells. Notice that few (<1%) active caspase-3^+^ cells contained P62^+^ inclusions (see Fig. S3D–F). Similar results were obtained by TUNEL staining with P62 co-immunostaining analysis (see Fig. S3D–F). Nearly none of the TUNEL^+^ cells had P62^+^ inclusions in the mutant cortex; or nearly all of the P62^+^ inclusion containing cells were negative for TUNEL (see Fig. S3D–F). These results support the view that autophagy and apoptosis are two different process [70, 71]. As Vps35/retromer regulates endosomal-to-Golgi trafficking of various transmembrane cargo proteins, it is likely to be involved in both autophagy and apoptosis pathways.

How does Vps35 dysfunction contribute to different neurodegenerative disorders? In light of our observations in various *Vps35*-cKO mouse models, we speculate that “when” and “where” the Vps35 is lost are crucial factors. *Vps35*-loss in embryonic cortical neurons during development may predispose to later development of FTD/AD, and Vps35-decrease in DA neurons in SNpc may predispose to a later development of PD [12]. Interestingly, a recent report that knocking down Vps35 in adult hippocampal neurons exhibit comparable dendritic spine/synaptic morphology with that of controls [72]. However, AD-relevant deficits, including impaired glutamatergic transmission and decreased long term potentiation are observed in these Vps35-deficient neurons [72, 73]. These different results are likely due to Vps35 functioning in an age and space dependent manners. For example, Vps35 may promote AMPA receptor surface targeting in adult hippocampal/cortical pyramidal neurons [72–74]. But, in embryonic/young cortical neurons, Vps35 appears to be critical for Sort1 trafficking. In DA neurons, Mul1, Drp1, and Lamp2a appear to be critical cargos of Vps35, contributing to Vps35-loss-induced PD pathogenesis [12–14]. Thus, at different age and in different type of cells, Vps35 regulates different cargos, therefore, involved in the development of different neurodegenerative disorders.

## Supplementary information

Supplemental Figure Legends

Supplemental Figure 1

Supplemental Figure 2

Supplemental Figure 3

Supplemental Figure 4

Supplemental Figure 5

Supplemental Figure 6

Supplemental Figure 7
