# Supplementary material for: Coupling of terminal differentiation deficit with neurodegenerative pathology in Vps35-deficient pyramidal neurons
Source: Cell Death Differ. 2020 Jan 6;27(7):2099–116. doi: 10.1038/s41418-019-0487-2 (PMC7308361; doi:10.1038/s41418-019-0487-2)
Supplement: Supplementary file 1 — Supplemental Figure Legends [file 41418_2019_487_MOESM1_ESM.docx]

**Supplemental Figure Legends**

**Fig S1.** **Generation of *Vps35^Neurod6^* mice. (A)** Postnatal growth retardation of *Vps35^Neurod6^* mice. **(B)** Body weight and Kaplan–Meier survival curves of *Vps35^Neurod6^* mice and littermate controls. **(C)** Representative images of immunostaining on the cortical L2-3 neurons of *Vps35^f/f^* (control) and *Vps35^Neurod6^* animals with Map2 (red) and Vps35 (green). The Map2^+^ cells lack Vps35 immunoreactivity in *Vps35^Neurod6^* animals, whereas these cells are Vps35 positive in Control animals. n=10. **(D)** Western blot analysis of Vps35 levels in different brain tissues taken from *Vps35^f/f^* and *Vps35^Neurod6^* animals. Gapdh was employed as a loading control.

**Fig S2.** **Golgi staining showed a decreased dendritic arborization and spine density *Vps35^Neurod6^* neocortex.** **(A)** Representative images of a Golgi stained pyramidal neuron from the L2-3 projection neurons. **(B)** Schematic representation of L2-3 pyramidal neurons that were traced in three dimensions and assessed by Sholl analysis. **(C-D)** Quantification shows a reduction of the apical and basal dendrites length (C), n=10 neurons from 3 mice per genotype; unpaired two-tailed t-test) and decreased dendritic complexity (D), by sholl analysis; n=3 neurons from 3 mice per group; two-way ANOVA with a Tukey’s multiple comparisons test) in the *Vps35^Neurod6^* mutants. **(E)** Representative image apical dendritic spines of cortical L2-3 pyramidal neurons. **(F)** Quantification of spine density in control and Vps35Neurod6 animals reveals significant differences between genotypes (n=10 neurons from 3 mice per genotype; two-tailed unpaired t-test). Scale bars: in A, E and H, 10μm and in D, 100 μm. *P < 0.05; ***P < 0.001; n.s., not significant.

**Fig S3 Apoptosis and DNA damage in *Vps35^Neurod6^* neocortex. (A and C)** Representative images of TUNEL assay, γH2AX and P53 staining. Immunostaining analysis using indicated antibodies in P21 neocortical sections. Higher-magnification images of the boxed regions were shown in left panels. **(B)** Quantification shows an increased TUNEL and DNA damage in *Vps35^Neurod6^* brains. n=3~4; unpaired two-tailed t-test. **(D-E)** Representative images of immunostaining analysis of P62 and apoptotic cells in P21 neocortical sections. **(F)** Quantification of percentage cells with indicated makers in *Vps35^Neurod6^* neocortex.

**Fig S4. Little effect on *Vps35*-KO-induced deficits by overexpression of Pgrn. (A, B)** Western blot and quantification analysis indicated the Pgrn levels were reduced in *Vps35^Neurod6^* neocortex. **(C, D)** Representative images from in utero electroporation experiments. Constructs were electroporated in *Vps35^f/f^* embryos at E14.5 before analysis at P14. Representative images of mouse brains (A) and single neuron (B) electroporated with either *CAG-GFP (Control)*, *CAG-Cre (Vps35-KO)* and *CAG-Cre with CAG-Pgrn (Vps35-KO+Pgrn OE)* vectors followed by immunostaining with Pgrn antibody. **(E-G)** Quantification of total dendritic length (E), spine density (F) and axonal spheroid size (G) reveals no significant difference when comparing *Vps35*-KO and *Vps35*-KO + *Pgrn* overexpression neurons. n=12. Scale bars: in C, 500 μm; and in D, 10 μm. Individual data points were shown as dots with group mean±s.e.m.

**Fig S5. Altered Sort1 distribution in Vps35-KO neurons. (A)** Western blot analysis of homogenates (by 1% TritonX-100 + 0.5% SDS) of neocortex by using the indicated antibodies. **(B)** Quantification analysiss of the relative protein expression levels in A. n=4. **(C)** Quantification of mRNA expression in the P1 forebrain. There was no significant difference when comparing *Vps35^Neurod6^* neocortex and littermate controls (n=3) except Vps35. **(D)** Primary cortical neurons (DIV10) from Vps35^f/f^ mice were transfected with BFP or Cre-BFP at DIV2. Upper panel: Cells were stained with GM130, Rab5, Lamp1 and Rab11 antibodies with Sort1 antibody. Lower panel: profile of fluorescence intensity of the white line in upper panel. **(E)** Mander’s colocalization coefficient reveals accumulation of Sort1 in lysosomes (n=6 neurons from 3 mice per genotype; two-tailed unpaired t-test). **(F)** Altered Sort1 distribution in neocortical neurons in *Vps35^Neurod6^* mice. Co-immunostaining analysis of Sort1 (green) and GM130/Lamp1 (red) in the L2-3 neurons. **(G)** Mander’s colocalization coefficient revealing accumulations of Sort1 in lysosomes of P14 *Vps35^Neurod6^* neurons (n=12 neurons from 4 mice per group, unpaired two-tailed t-test). **(H)** Western blot analyses of total, surface and lysosomal protein extracts from control and Vps35Neurod6 neocortex at P1 using indicated antibodies. **(I)** Quantification analysis of data in H. (n=4 mice per group, unpaired two-tailed t-test). Scale bars: in D and F, 10 μm. Individual data points were shown as dots with group mean±s.e.m.

**Fig S6. Partial rescue of the degeneration phenotype by suppression of Sort expression in *Vps35^Neurod6^* mice. (A)** Western blot analysis of Sort1 in Neuro2a cells transfected with shRNAs targeting Sort1 (*Sort1* shR 2# and 3#) or a scrambled negative control (Mock shR). Quantification analysis (in lower panel) revealed a 38% reduction in Sort1 protein levels following transfection with a *Sort1* shR 2# and a 68% reduction by Sort1 shR 3#. n=3. **(B)** Representative images of P14 cortical neurons which were electroporated with shRNAs targeting Sort1 (Sort1 shR 2# and 3#) or a scrambled negative control (Mock shR) staining with Sort1 antibody indicate that the shR-Sort1 (particularly #3) efficiently suppressed the expression of Sort1 *in vivo.* **(C and D)** Representative images of neurons electroporated with either *Mock* shR or *Sort1* shR into *Vps35^f/f^* or *Vps35^Neurod6^* followed by immunostaining with GFP antibody. Constructs were electroporated at E14.5 before analysis at P14. **(E-G)** Quantification of total dendritic length (E), spine number (F) and axonal spheroid size (G) and reveals a partially rescue degeneration phenotype of the neuron electroporation with *Sort1* shR in *Vps35^Neurod6^* mice (n=10 neurons from 3 mice per group, one-way ANOVA with Tukey’s multiple comparison test).

**Fig S7. A working model showing Vps35/retromer’s function in mouse embryonic pyramidal (Py) neurons to promote axon and dendrite terminal differentiation and to prevent FTD pathogenesis.**
