# Supplementary figures and images for: Coupling of terminal differentiation deficit with neurodegenerative pathology in Vps35-deficient pyramidal neurons
Source: Cell Death Differ. 2020 Jan 6;27(7):2099–116. doi: 10.1038/s41418-019-0487-2 (PMC7308361; doi:10.1038/s41418-019-0487-2)

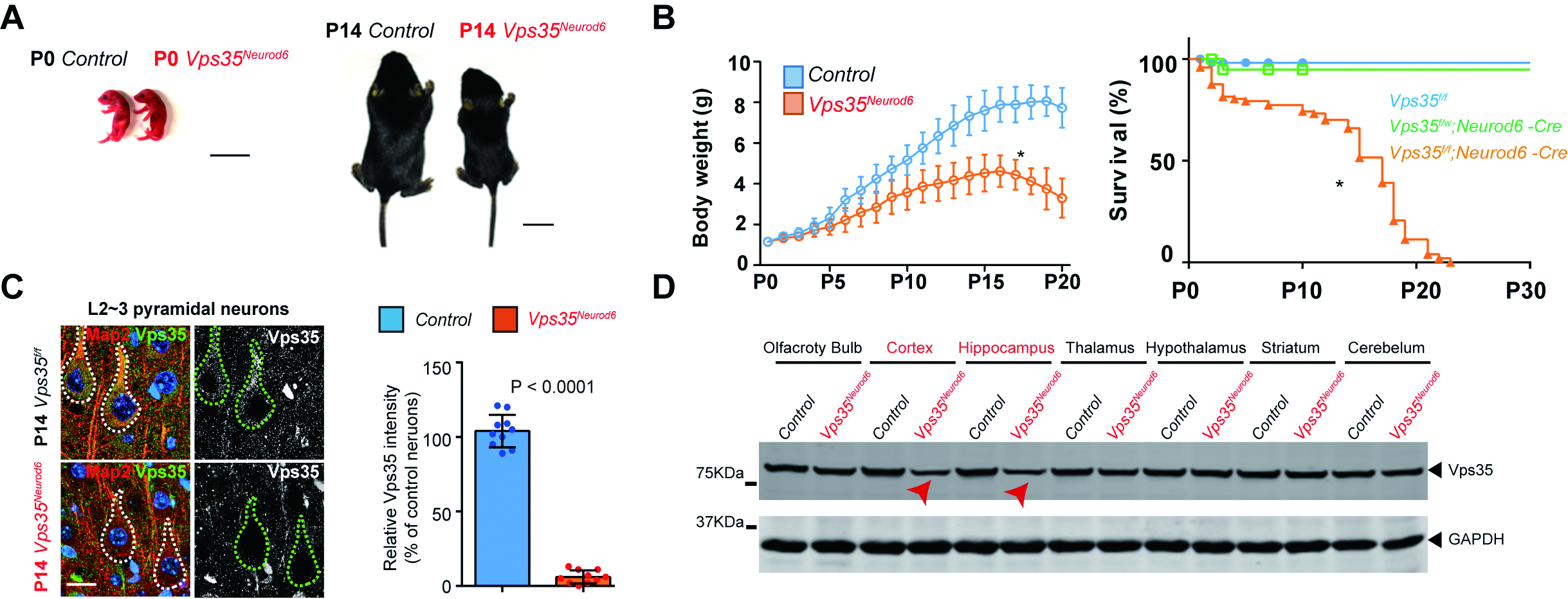

Supplement: Supplementary file 2 — Supplemental Figure 1 [file 41418_2019_487_MOESM2_ESM.tif]

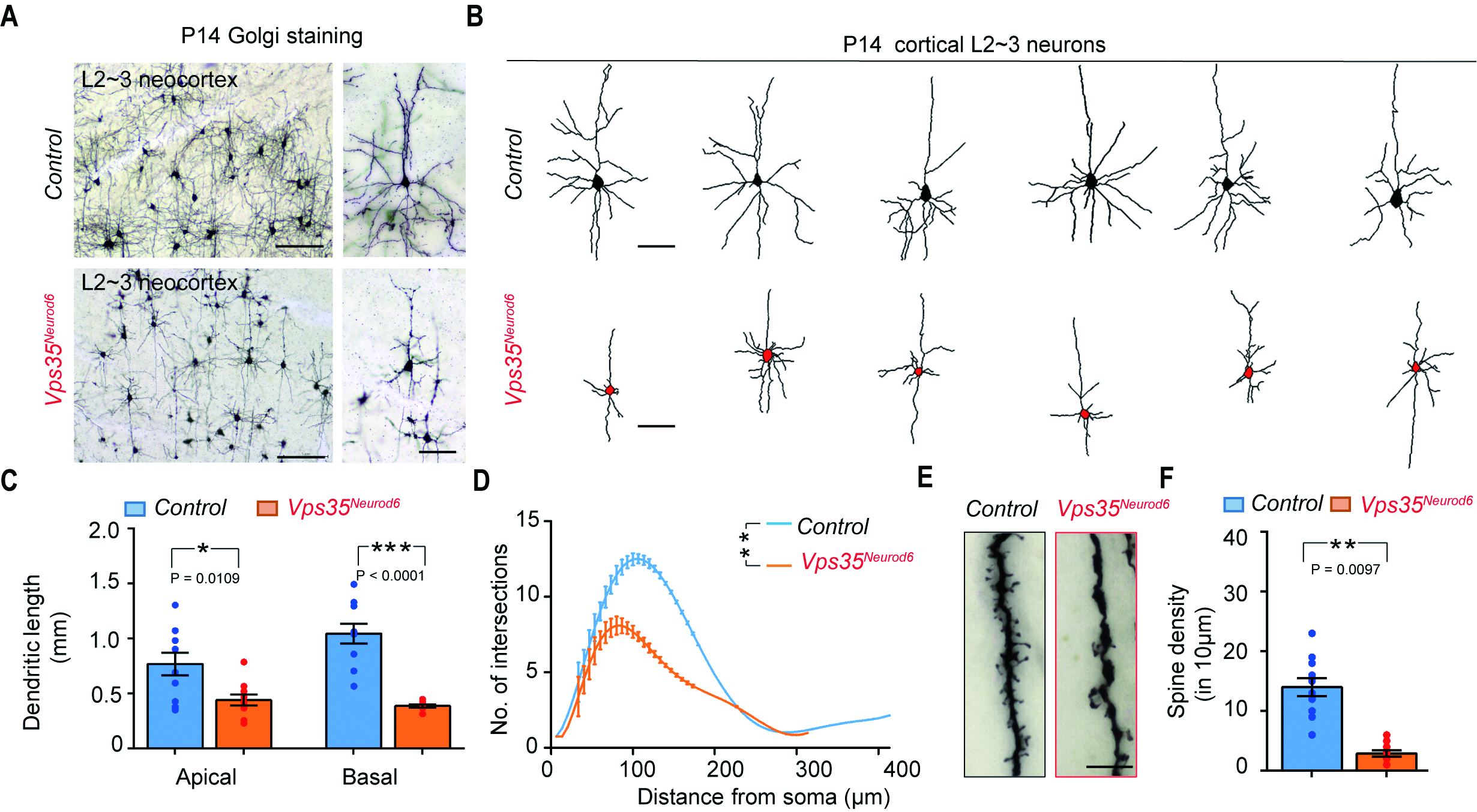

Supplement: Supplementary file 3 — Supplemental Figure 2 [file 41418_2019_487_MOESM3_ESM.tif]

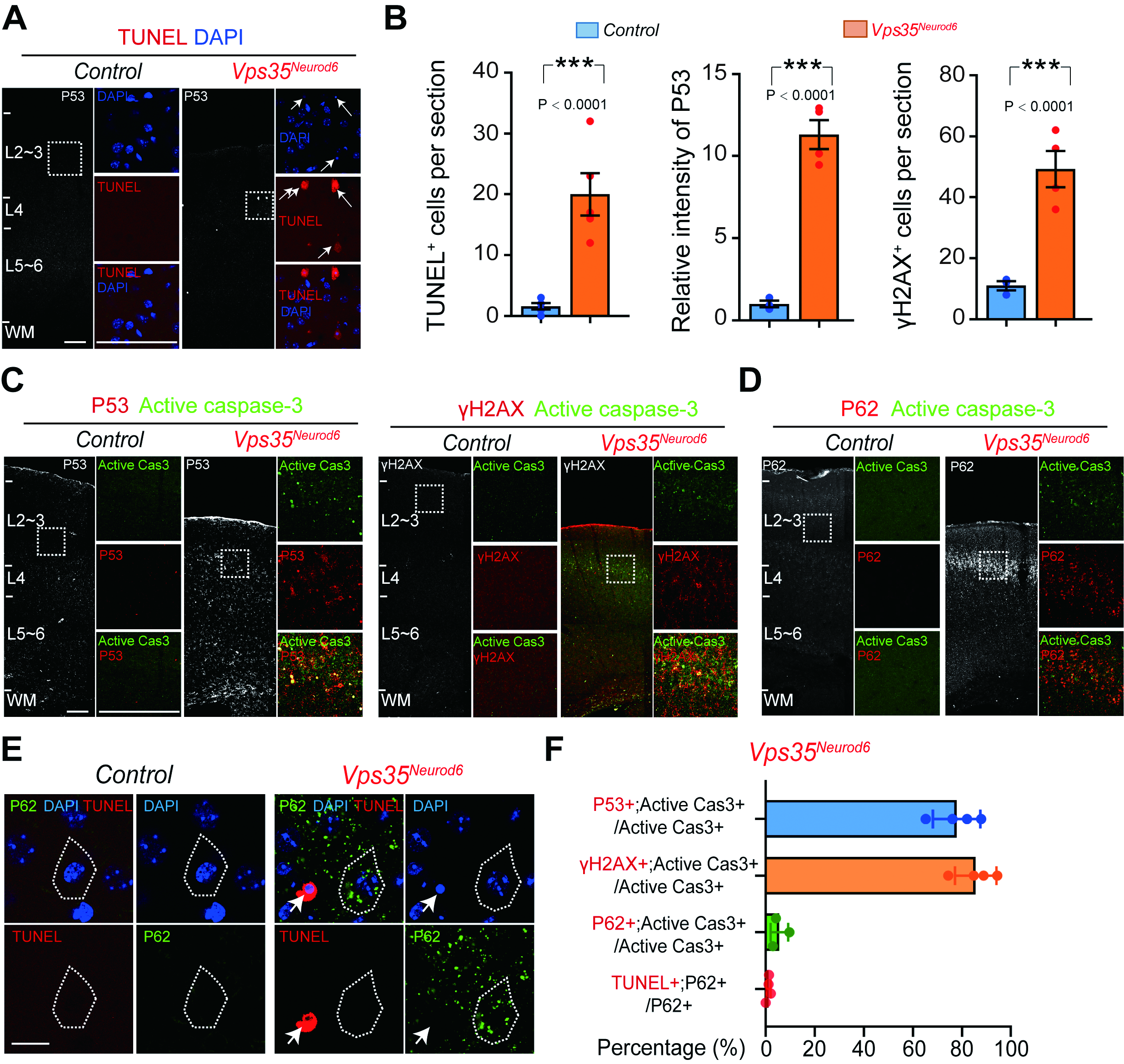

Supplement: Supplementary file 4 — Supplemental Figure 3 [file 41418_2019_487_MOESM4_ESM.tif]

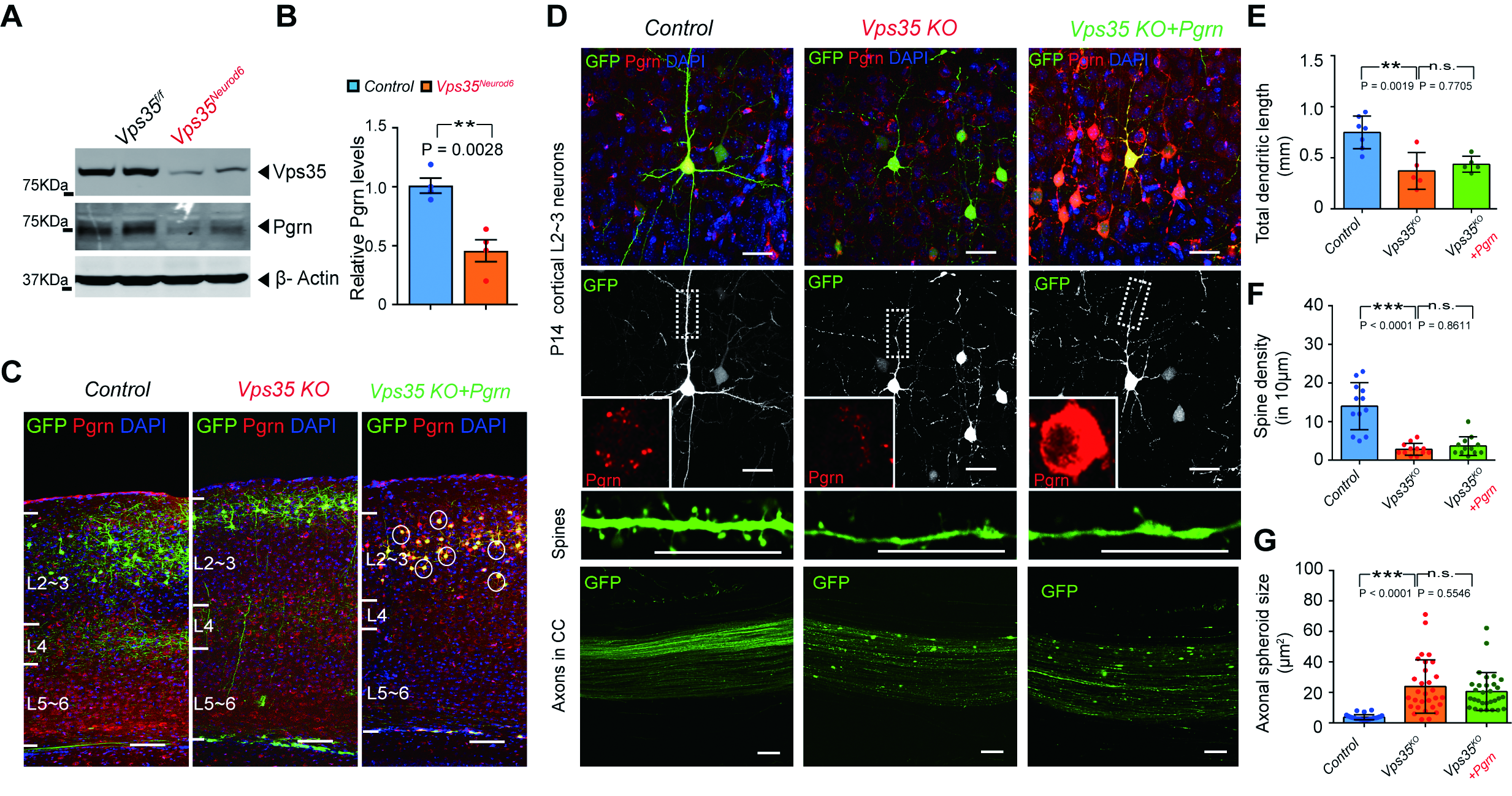

Supplement: Supplementary file 5 — Supplemental Figure 4 [file 41418_2019_487_MOESM5_ESM.tif]

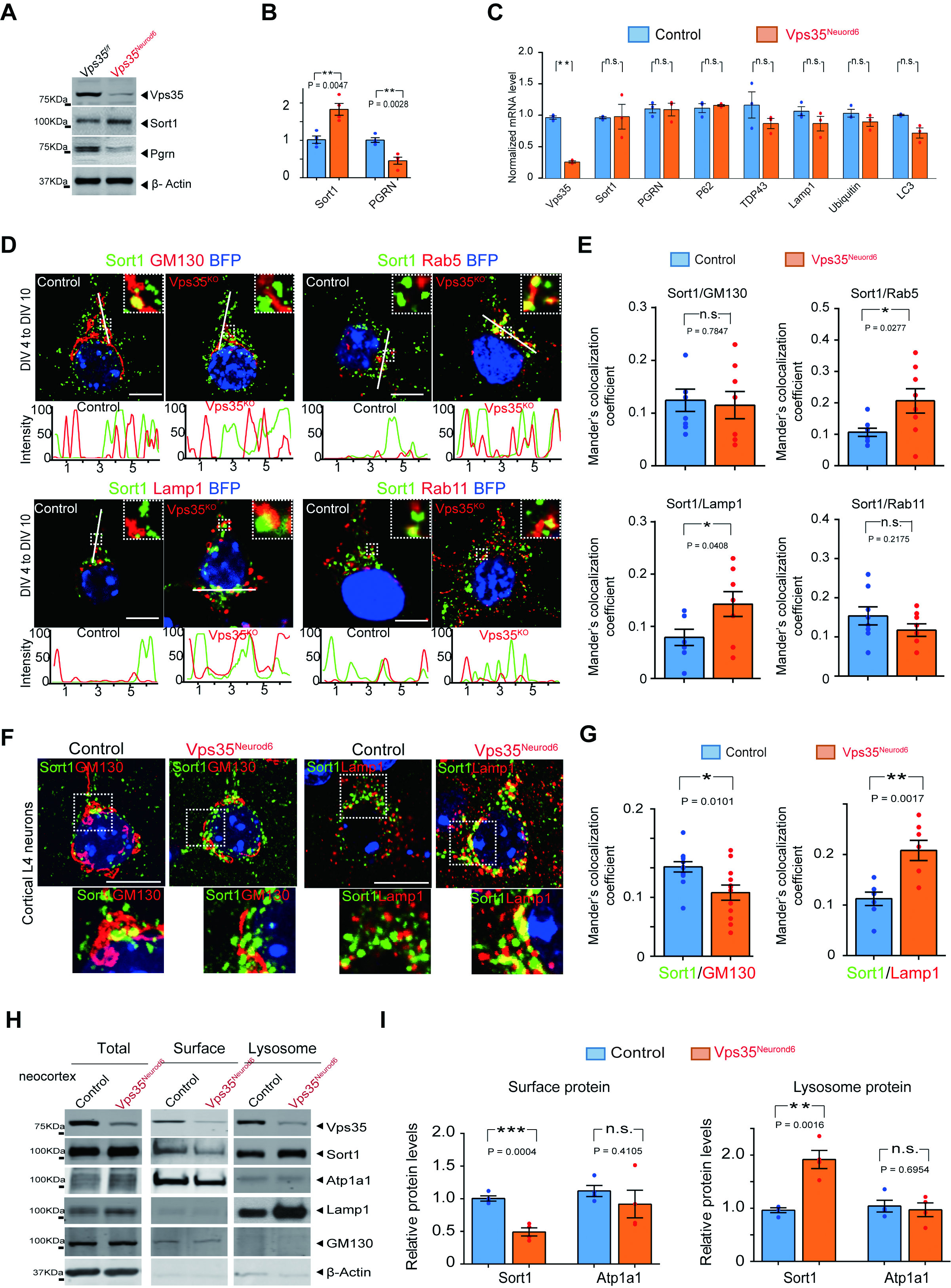

Supplement: Supplementary file 6 — Supplemental Figure 5 [file 41418_2019_487_MOESM6_ESM.tif]

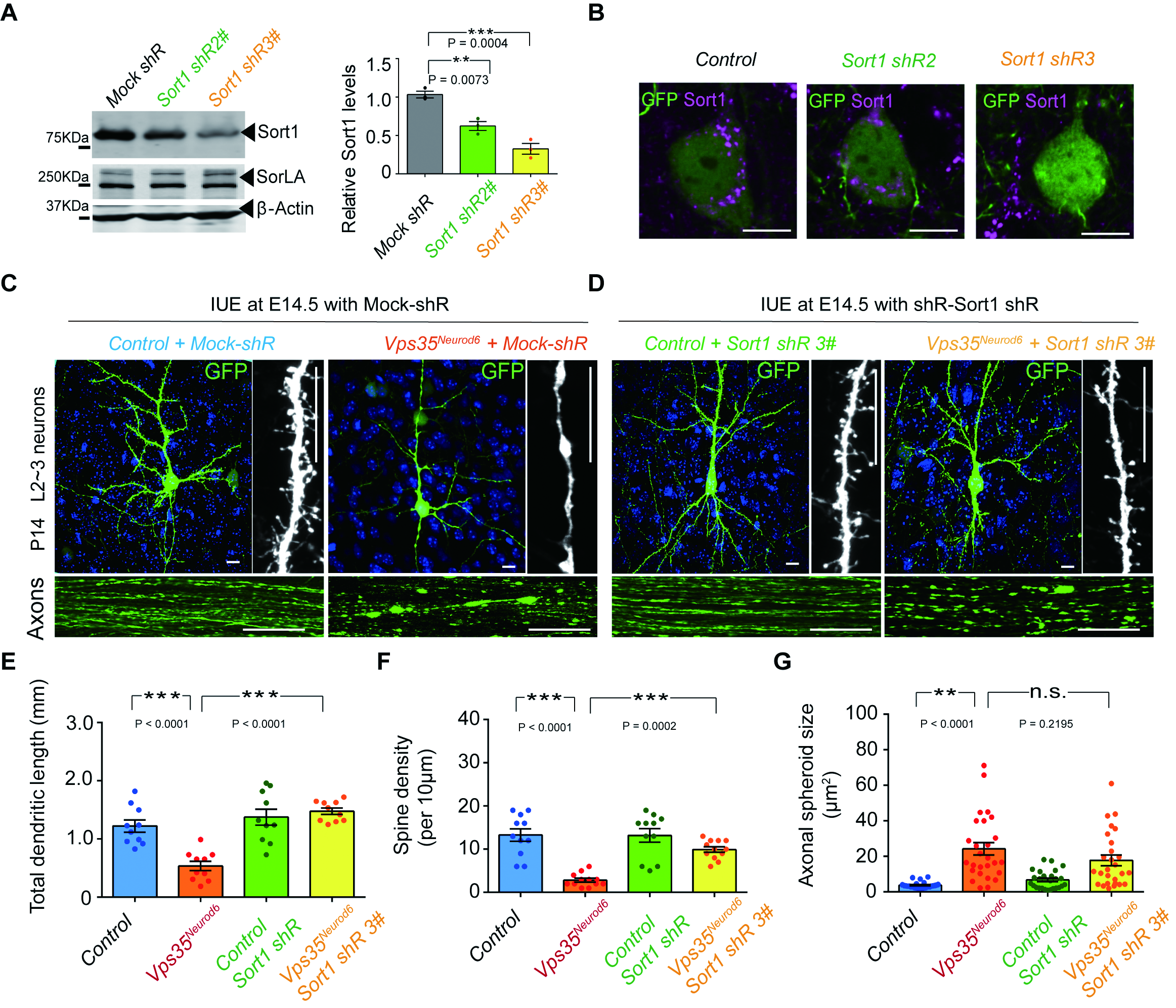

Supplement: Supplementary file 7 — Supplemental Figure 6 [file 41418_2019_487_MOESM7_ESM.tif]

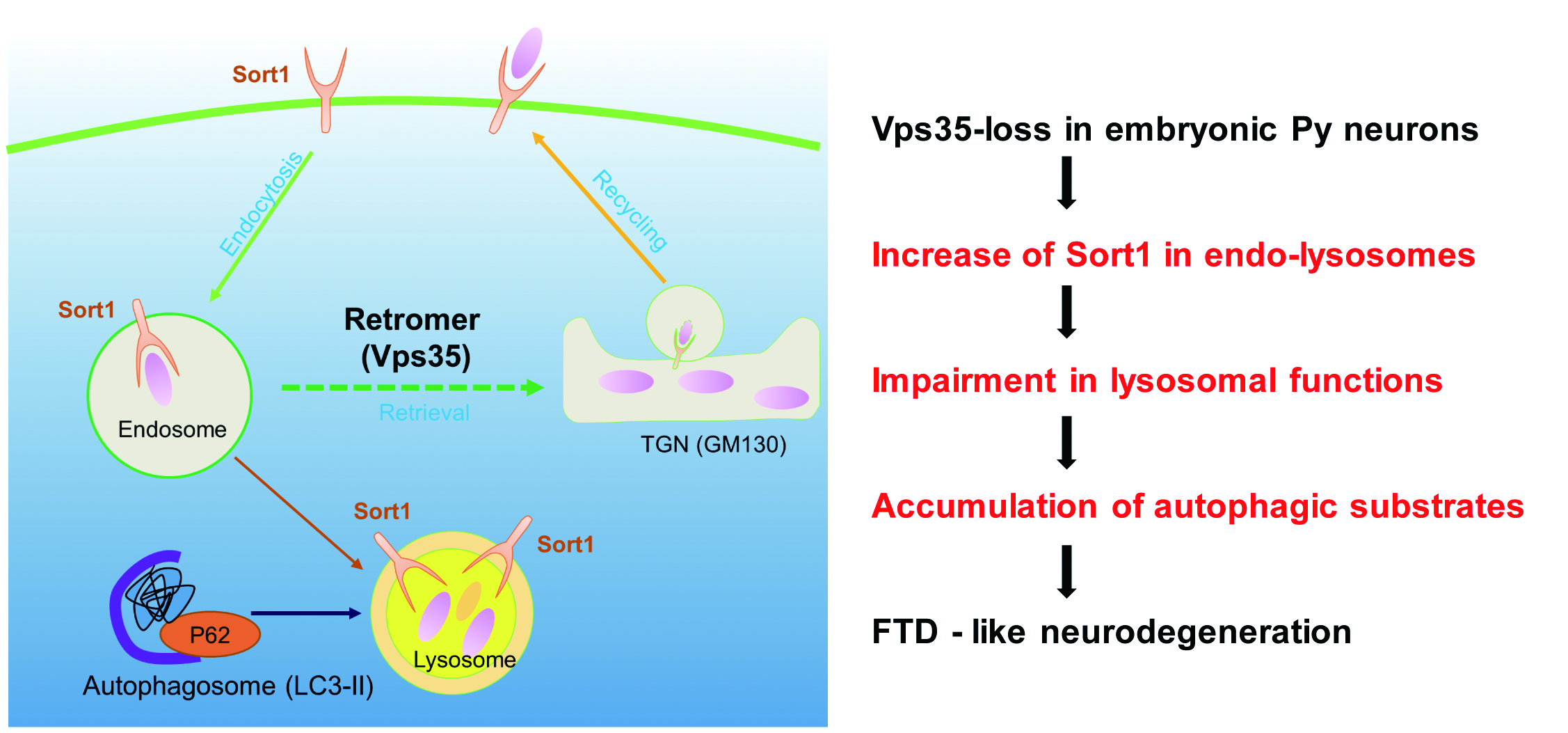

Supplement: Supplementary file 8 — Supplemental Figure 7 [file 41418_2019_487_MOESM8_ESM.tif]
